# Exosome-bearing hydrogels and cardiac tissue regeneration

**DOI:** 10.1186/s40824-023-00433-3

**Published:** 2023-10-06

**Authors:** Hassan Amini, Atieh Rezaei Namjoo, Maryam Taghavi Narmi, Narges Mardi, Samaneh Narimani, Ozra Naturi, Nafiseh Didar Khosrowshahi, Reza Rahbarghazi, Solmaz Saghebasl, Shahriar Hashemzadeh, Mohammad Nouri

**Affiliations:** 1 Stem Cell Research Center, Tabriz University of Medical Sciences, Tabriz, Iran; 2 Department of General and Vascular Surgery, Tabriz University of Medical Sciences, Tabriz, 51548/53431 Iran; 3 Drug Applied Research Center, Tabriz University of Medical Sciences, Tabriz, Iran; 4 Student Research Committee, Tabriz University of Medical Sciences, Tabriz, Iran; 5 Department of Organic and Biochemistry, Faculty of Chemistry, University of Tabriz, Tabriz, Iran; 6 Stem Cell and Tissue Engineering Research Laboratory, Sahand University of Technology, Tabriz, 51335-1996 Iran; 7 Department of Applied Cell Sciences, Faculty of Advanced Medical Sciences, Tabriz University of Medical Sciences, Tabriz, 51548/53431 Iran

**Keywords:** Cardiac tissue engineering, Infarction, Exosomes, Hydrogels, Encapsulation, Regeneration

## Abstract

**Background:**

In recent years, cardiovascular disease in particular myocardial infarction (MI) has become the predominant cause of human disability and mortality in the clinical setting. The restricted capacity of adult cardiomyocytes to proliferate and restore the function of infarcted sites is a challenging issue after the occurrence of MI. The application of stem cells and byproducts such as exosomes (Exos) has paved the way for the alleviation of cardiac tissue injury along with conventional medications in clinics. However, the short lifespan and activation of alloreactive immune cells in response to Exos and stem cells are the main issues in patients with MI. Therefore, there is an urgent demand to develop therapeutic approaches with minimum invasion for the restoration of cardiac function.

**Main body:**

Here, we focused on recent data associated with the application of Exo-loaded hydrogels in ischemic cardiac tissue. Whether and how the advances in tissue engineering modalities have increased the efficiency of whole-based and byproducts (Exos) therapies under ischemic conditions. The integration of nanotechnology and nanobiology for designing novel smart biomaterials with therapeutic outcomes was highlighted.

**Conclusion:**

Hydrogels can provide suitable platforms for the transfer of Exos, small molecules, drugs, and other bioactive factors for direct injection into the damaged myocardium. Future studies should focus on the improvement of physicochemical properties of Exo-bearing hydrogel to translate for the standard treatment options.

**Graphical Abstract:**

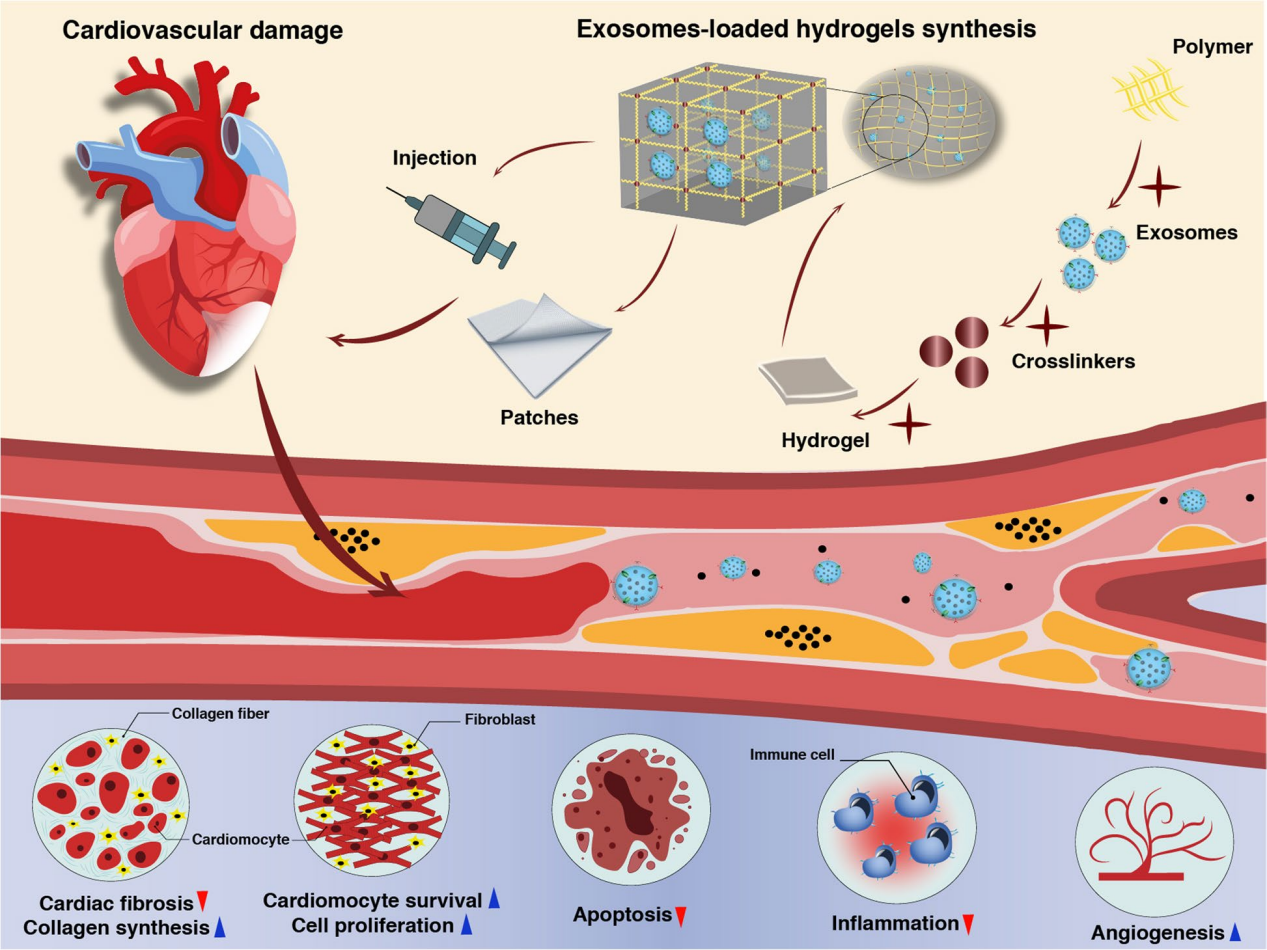

## Introduction

It has been well-established that a small fraction of injured cardiomyocytes is replaced following pathological conditions especially myocardial infarction (MI) due to the minimum regenerative potential of surrounding healthy cardiomyocytes [1]. Thus, myocardial regeneration is a challenging issue in the clinical setting. Along with several medication strategies, cell transplantation using mature and stem cell types has been developed for the restoration of injured myocardium and replacement of damaged cells [2] (Table 1). In contrast to the systemic approach, direct injection of stem cells into the infarcted myocardium is an effective strategy to deliver an approximately appropriate number of cells into the damaged area without the need for target delivery and homing [3].

**Table 1:** Completed clinical trials evaluating different stem cells for myocardial ischemia (MI)

Clinical trial	Completed date	Cell source	Delivery pathway	Outcome(s)
TOPCARE-AMI [4]	2002	Peripheral progenitor cells/bone marrow MSCs	Intracoronary	Ejection fraction↑, infarct size↓
TOPCARE-AMI [5]	2004	Peripheral progenitor cells/ bone-marrow MSCs	Intracoronary	LVEF↑, infarct size↓
BOOST [6]	2004	Autologous bone marrow MSCs	Intracoronary	LVEF↑
MAGIC [7]	2004	Peripheral progenitor cells	Intracoronary	Myocardial perfusion↑, LVEF↑
IACT [8]	2005	Autologous mononuclear bone marrow cells	Intracoronary	LVEF↑, infarct size↓, wall movement velocity↑
Acute Myocardial Infarction [9]	2006	Autologous bone marrow MSCs	Intracoronary	Exercise time↑, peak heart rate↑
REPAIR-AMI [10]	2006	Autologous bone marrow MSCs	Intracoronary	LVEF↑, infarct size↓, revascularization↑
HEBE [11]	2007	Peripheral progenitor cells/bone marrow MSCs	Intracoronary	Non-significant changes in left ventricular volumes, mass, and infarct size
MYSTAR [12]	2008	Autologous bone marrow MSCs	Intra-myocardial and intracoronary	LVEF↑, infarct size↓
REGENT [13]	2008	Autologous bone marrow MSCs	Intracoronary	No significant improvement in LV ejection fraction
Patients with acute MI [14]	2009	Autologous bone marrow MSCs	Intra-myocardial	LVEF↑, infarct size↓
SEED-MSC [15]	2010	Autologous bone marrow MSCs	Intracoronary	LVEF↑
Apollo [16]	2010	Adipose tissue MSCs	Intracoronary	No significant improvement in LV ejection fraction and infarct size
CADUCEUS [17]	2011	Autologous cardiosphere-derived stem cells	Intracoronary	Infarct size↓
Patients with acute MI [18]	2012	Human Wharton’s jelly-derived MSCs	Intracoronary	LVEF↑, infarct size↓
The Late TIME Study [19]	2012	Autologous bone marrow mononuclear cells	Intracoronary	No significant improvement in LV ejection fraction and infarct size
COMPARE CPM-RMI [20]	2012	Autologous bone marrow CD133^+^ cells	Intra-myocardial	LVEF↑, infarct size↓, viability↑
SCAMI [21].	2013	Autologous bone-marrow MSCs	Intracoronary	LVEF↑
SCIPIO [22]	2013	Cardiac stem cells	Intracoronary	LVEF↑, infarct size↓
REPAIR-AMI [23]	2014	Autologous bone marrow MSCs	Intracoronary	Unknown death or rehospitalization↓
MyStromalCell [24]	2014	Autologous VEGF-A165-stimulated adipose tissue MSCs	Intra-myocardial	No significant outcome
PoseidonDCM [25]	2016	Autologous and allogeneic bone-marrow MSCs	Transendocardially	Cardiac function↑ in the allogeneic-treated group
CAREMI [26]	2016	Allogeneic human cardiac stem cells	Intracoronary	No significant improvement in LV volumes, ejection fraction, or regional wall motion
PreSERVE-AMI [27]	2017	Autologous bone marrow MSCs	Intracoronary	LVEF↑, infarct size↓
Patients with elevated ST [28]	2018	Autologous bone marrow mononuclear cells	Intracoronary	No significant improvement in LVEF and infarct size

Among different stem cell types, mesenchymal stem cells (MSCs) are the main cell source used for the healing of injured myocardium [29]. These cells can be isolated and expanded easily from different tissues without the necessity of specific conditions that are required for the cultivation of other cell types [30]. Due to its appropriate differentiation properties toward cardiovascular cells, and anti-fibrotic and immunomodulatory properties with angiogenic potential, the application of MSCs is at the center of attention for several pathological conditions occurring within the cardiac tissue [31]. Along with the differentiation of MSCs into cardiomyocytes, the release of cytokines and growth factors via paracrine manner can also promote the regeneration of injured cardiac tissue [32]. Upon the administration of MSCs into the ischemic cardiac tissue, the function of the left ventricle is improved because of the stimulation of cardiomyocyte proliferation, angiogenesis, and inhibition of oxidative stress, excessive immune cell responses, and fibrosis [33].

Regardless of cell type and the injection method, the regenerative outcome and restoration of cardiac tissue function are not satisfactory because of the low-rate engraftment capacity of transplant cells after administration into the target sites [34]. Thus, the most of studies related to cardiac cell therapy are restricted to experimental evaluation in animal models and clinical trials [35]. Of note, most of the blinded randomized trials have indicated uncertainty and inconsistent data in the improvement of cardiac tissue function [36]. Besides, strategies based on the administration of whole-cell therapies face some limitations that restrict the application of cells for different regenerative purposes. A large fraction of cells should be prepared before injection into the target site which needs prolonged culture and several passages [37]. These features can increase the probability of infections, and morphological and genetic alterations in in vitro setting [38]. Of note, administrated cells can be also eliminated because of the lack of an appropriate microenvironment, excessive mechanical stress during the administration, and activation of all-reactive immune cells [39].

In comparison with whole-cell-based therapies, the application of cell secretory vesicles like Exos is at the center of the debate. It was suggested that Exos are more stable for long periods after introduction into the target sites and exhibit appropriate biodistribution and immune tolerability [40]. Based on previous studies, Exos from both allogeneic and xenogeneic sources can be applied for the alleviation of several pathologies with the minimum immune cell response compared to whole cell-based therapies [41]. The existence of several uptake mechanisms such as direct fusion, ligand-based, clathrin- and caveolin-mediated endocytosis, macropinocytosis, and phagocytosis for Exos can increase delivery outcomes into the target sites [42]. Exos can harbor different therapeutic molecules such as proteins, lipids, and nucleic acids with the ability to restore the function of the injured myocardium. Besides, factors related to the modulation of apoptosis, fibrosis, and angiogenesis are normally present in exosomal cargo [43]. Activation of certain signaling pathways inside the injured cardiomyocytes such as ERK1/2, p38MAPK, and TLR4 can increase cell resistance against the insulting conditions. These features make Exos as valid therapeutic tool for the acceleration of the healing process in injured myocardium [44].

Despite putative advantages, the application of Exos in regenerative medicine encounters some fundamental limitations [38]. At present, several isolation methods are used for the isolation and purification of Exos from different sources [45]. Most conventional isolation methods are expensive, laborious, and time-consuming without standard guidelines [46]. The systemic (intravenous) administration, subcutaneous, and intraperitoneal injections of Exos can increase the possibility of off-target delivery and leads to inappropriate delivery of Exos into the injured myocardium [47]. Using a sophisticated imaging system, the most fraction of administrated Exos are eliminated by the reticuloendothelial system located in hepatic and splenic tissues [3]. Surface modifications and different ex vivo manipulations can alter the physicochemical properties of Exos because of the formation of protein corona and absorbance of the external protein layer [48]. Commensurate with these descriptions, the advent and development of *de novo* delivery approaches are mandatory to yield better regenerative outcomes.

In recent decades, the application of hydrogels has been increased for in situ delivery of stem cells and nano-sized particles like Exos into the injured sites [33]. It is postulated that hydrogels provide an extracellular matrix (ECM)-like milieu with relatively similar physicochemical values while can control the recruitment and activity of different immune cell types [49]. Compared to several synthetic nanocarriers, Exos with appropriate biocompatibility, less toxicity, immunogenicity, and stability are at the center of debate for cardiovascular diseases [50]. Exo-loaded hydrogels could be considered a relevant innovative treatment strategy for the regeneration and functional improvement of injured myocardium. The incorporation of Exos with biomaterial-based hydrogels creates an appropriate supporting niche for sustained release that can compensate for the drawbacks associated with direct Exo administration [51]. Here, in this review article, recent advances in the application of Exo-loaded hydrogels were discussed in terms of cardiac tissue injuries.

## Exo biogenesis

**Fig. 1:**
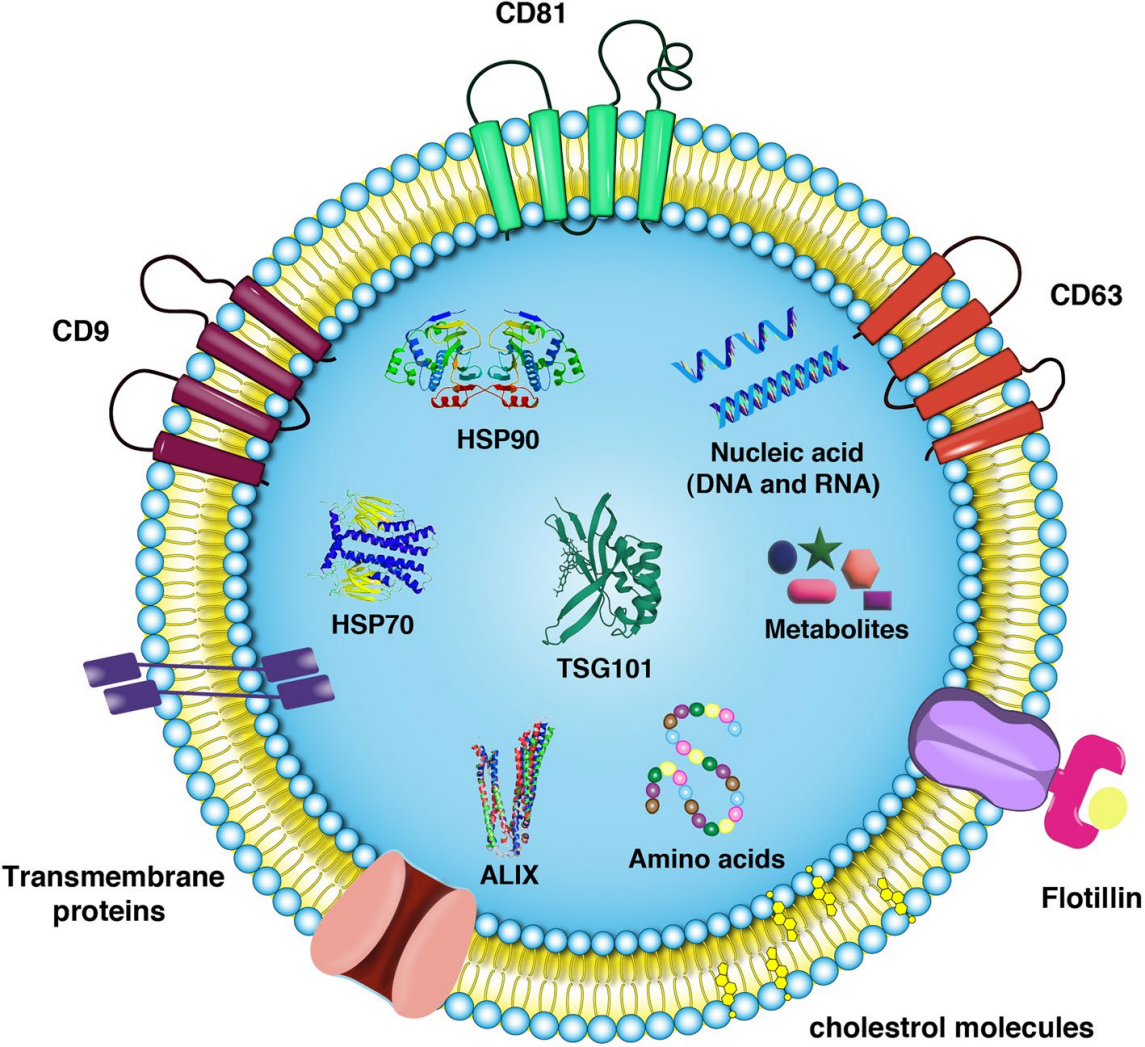
The structure of exosomes (Exos) with the lipid bilayer. Exos can transfer specific cargo (protein and genetic contents) from donor cells to recipient cells in a paracrine manner. The type and levels of specific signaling molecules can induce/inhibit certain molecular pathways inside the target cells

In multicellular organisms, intercellular communication can be done in a paracrine manner via the unidirectional or bidirectional transfer of signaling molecules in addition to close physical cell–to–cell connection [52]. To date, different studies have highlighted the critical role of extracellular vesicles (EVs) including exosomes (Exos), microvesicles, and apoptotic bodies in paracrine interaction between the cells. Exos are important EV types with an average diameter of 30–150 nm and circulate in biofluids [53]. These nano-sized vesicles can easily transfer the signaling biomolecules, cytokines, and other compounds from donor cells to recipient cells and can indicate the real-time proteomic and genomic changes in the parent cells (Fig. 1) [53]. Exos are produced by the activity of several factors belonging to the endosomal system where several signaling molecules are sequestrated into the exosomal lumen [54]. The phenomenon of cargo transfer from one cell to another cell can contribute to the modulation of numerous biological events. In this regard, Exos can partake in antigen presentation [55], coagulation [56], proliferation [57], differentiation [58, 59], immune cell signaling [60], angiogenesis [61–63], wound healing [64, 65], regeneration [66], growth and organ development [67, 68], and pathological changes [69–71]. These features indicate that Exos can be considered a theranostic tool in several pathologies [53, 71–74].

**Fig. 2:**
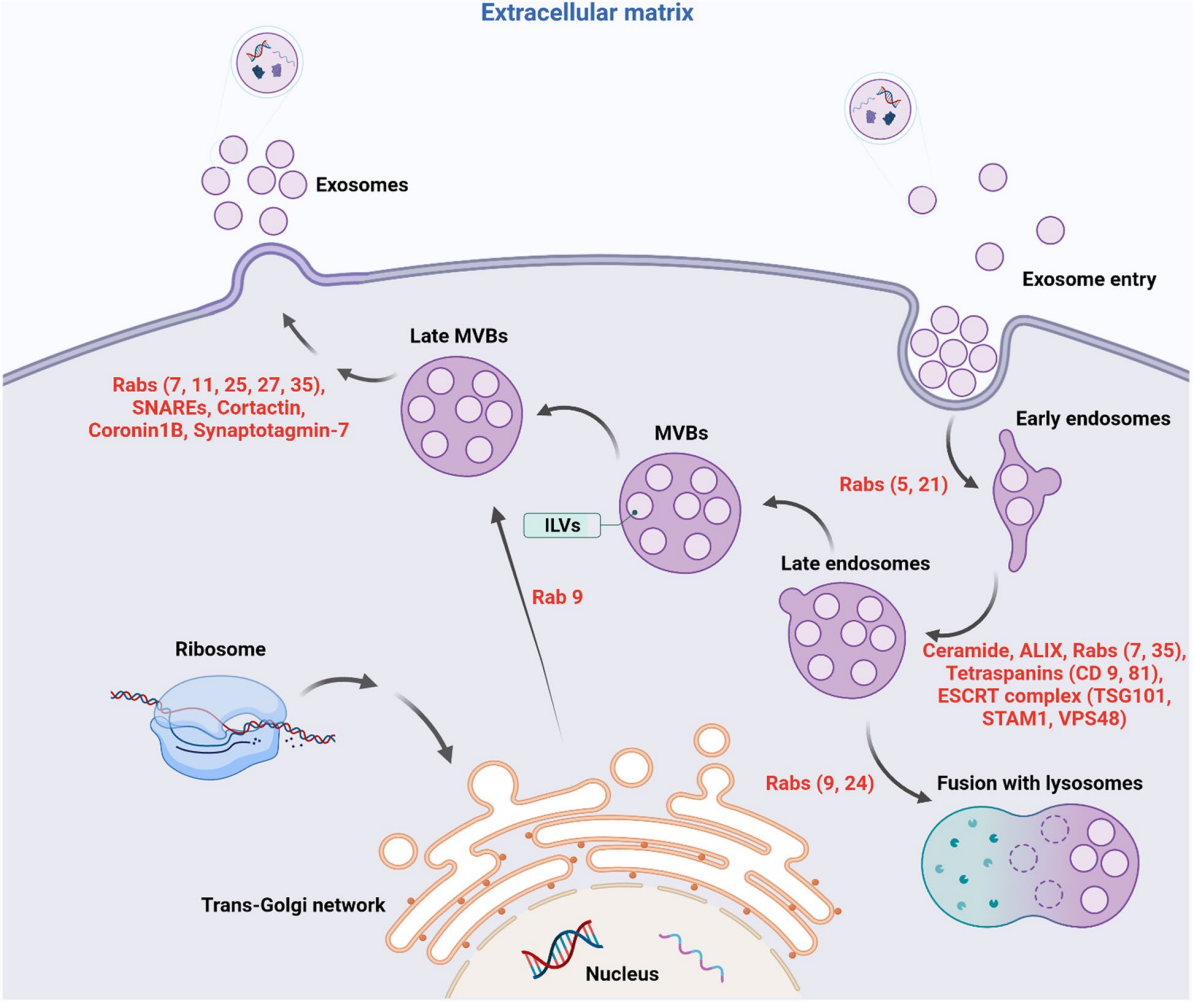
Molecular pathways associated with Exo generation and release. An endosomal system composed of early, and late endosomes and multi-vesicular bodies (MVBs) plays a key role in the production of Exos. Inside the endosomes, numerous ILVs are formed by the invagination of the membrane. Upon secretion into the ECM, ILVs are named Exos. Different factors such as ESCRT complex (ESCRT-0, -I, -II, and -III) and tetraspanins sequestrate the signaling molecules into the ILVs. In the latter steps, the SNARE system in collaboration with several GTPases directs the MVBs toward the cell membrane to release the Exos

Upon the activation of the endosomal system, the membrane of early and late endosomes is invaginated into the lumen, leading to the formation of numerous intraluminal vesicles (ILVs). In the latter steps, late endosomes mature into multivesicular bodies (MVBs) [75]. Simultaneous with the invagination of the endosomal membrane, several signaling molecules are ubiquitinated and directed into the ILV lumen in ESCRT-dependent and ESCRT-independent manner [76]. ESCRT machinery is composed of four complexes, 0, -I, -II-, and –III with associated factors such as VPS4, VTA1, and ALIX [77]. Along with this system, the Syndecan-Syntenin-Alix-ESCRT axis is thought of as an accessory ESCRT pathway involved in the sorting of un-ubiquitinated target molecules into ILVs [78, 79]. Likewise, the ESCRT-independent pathway composed of neutral sphingomyelinase 2 (nSMase 2) and tetraspanins also sorts the un-ubiquitinated cargos into the ILVs [79–81]. It is suggested that tetraspanins such as CD63, CD9, and CD81, etc. possess membrane microdomains and accelerate the ILV budding into the lumen of endosomes (Fig. 2) [79]. The conversion of sphingomyelin to ceramides by nSMase 2 activity is associated with the sequestration of flotillins and LC3 into the ILVs [82, 83]. Following the formation of ILVs and maturation of endosomes, the participation of GTPases belonging to the Rab family can transfer the endosomes and toward trans-Golgi apparatus (Rab9) [84, 85], lysosomes (Rab7) [86, 87], and cell membrane Rab27a and b [88–90] where tethering and the physical contact of MVBs with cell membrane leads to the release of ILVs into ECM where they are hereafter known as Exos [83]. Other Rabs such as Rab11 and Rab35 are involved in the endosomal recycling pathway [91]. The recruitment of Rab11, Rab35, and Rab27 can contribute to the stabilization of actin and activation of the SNARE complex which promotes the MVB-cell membrane fusion [70].

Despite the advantage of Exo application in regenerative medicine, several issues such as isolation modalities and purity rate limit the extensive application of Exos for the alleviation of different pathological conditions. For instance, it is highly recommended to isolate pure, homogeneous Exo pellets by using standard methods [92]. Several isolation methods such as differential magnetically activated cell sorting, ultracentrifugation, density gradient centrifugation, ultrafiltration, immunoaffinity capture, size-exclusion chromatography, microfluidics, and polymer-based precipitation have been used until yet [92–94]. Most of the exosomal separation approaches are based on physical and chemical properties like size, shape, charge, density, and composition of the membrane surface. It is thought that the sample source and isolation method can affect the purity of the final Exos. Besides, most conventional methods alter the exosomal morphology, size, content, and surface markers [95–97] (Table 2). Moreover, The stability and retention of Exos are the main obstacles to clinical applications [98]. They are cleared rapidly through the immune system or accumulate in the different organs and tissues like the liver, spleen, and lungs through blood circulation in vivo [99]. On the other hand, the difficulty and high cost of their purification prevent their usage in high doses at the site of interest [100]. To overcome limitations associated with the short half-life and maintain the bioactivity of Exos, combining Exos with different biomaterials such as hydrogels has become a considerable interest of investigation on Exo-based treatments [101]. Embedding Exos in a composite system increases their durability and stability and can control their delivery kinetics according to the ideal release schedule [102]. In addition, it creates an appropriate extracellular environment for the adhesion, proliferation, and migration of target cells [33].

**Table 2:** Several methods for the isolation of Exos

Isolation methods	Mechanism of Isolation	Advantages	Disadvantages
Differential ultracentrifugation	Size, and shape	The large content of Exos, low cost, absence of chemical reagents, and high quality (gold standard)	Time-consuming, not suitable for a small volume of biomaterial, isolation based on sedimentation rates, shape can deform
Density gradient centrifugation	Density, and size	single centrifugation step,	The time of centrifugation should be optimized. prolonged centrifugation leads to sedimentation of all components, low-productive, size-separation methods need to purify Exos, and shape can deform
Isopycnic gradient centrifugation	floatation densities	Single centrifugation step, particles stop in the final position	Time-consuming, low-productive, size-separation methods need to purify exosomes, shape can deform
Ultrafiltration	Size	Low consumption, high output, convenience, the integrity of exosomes, gradual removal of particles	Particles may attach to the filter membrane, Low purity, shape can deform
Immunoaffinity capture	Exosomal surface markers	High specificity and purity, low contamination	Expensive, time-consuming
Magnetically activated cell sorting (MACS)	Charge, magnetic beads	a quick, reliable method, with high purity, may be used for clinical approaches	Relatively expensive and time-consuming
Size-exclusion Chromatography	Size, weight, hydrodynamic radius	High purity, fast	Low purity and contamination with other molecules, low yield, the influence of external force
Microfluidics	Physicochemical properties	Automated, fast, cheap	No standardizing, no clinical
Polymer-based precipitation	Exo aggregation in the presence of precipitating agents	High functional and morphological quality, large volumes of biomaterial, much cheaper	Low purity, lack of isolation from other components, poor solubility of precipitated aggregates

## Biomaterials and myocardial regeneration

In recent years, we witnessed progress in cardiac tissue engineering to overcome limitations associated with whole-cell-based therapy approaches [103]. Cardiac tissue engineering aims to apply natural, synthetic, and semisynthetic biomaterials with multiple growth factors to improve the delivery outcome, survival rate, and function of transplant cells into infarcted sites [104]. Considering the distinct physicochemical properties of cardiac tissue, the development, and selection of suitable biomaterials for cardiac regeneration are of great importance [105, 106].

**Fig. 3:**
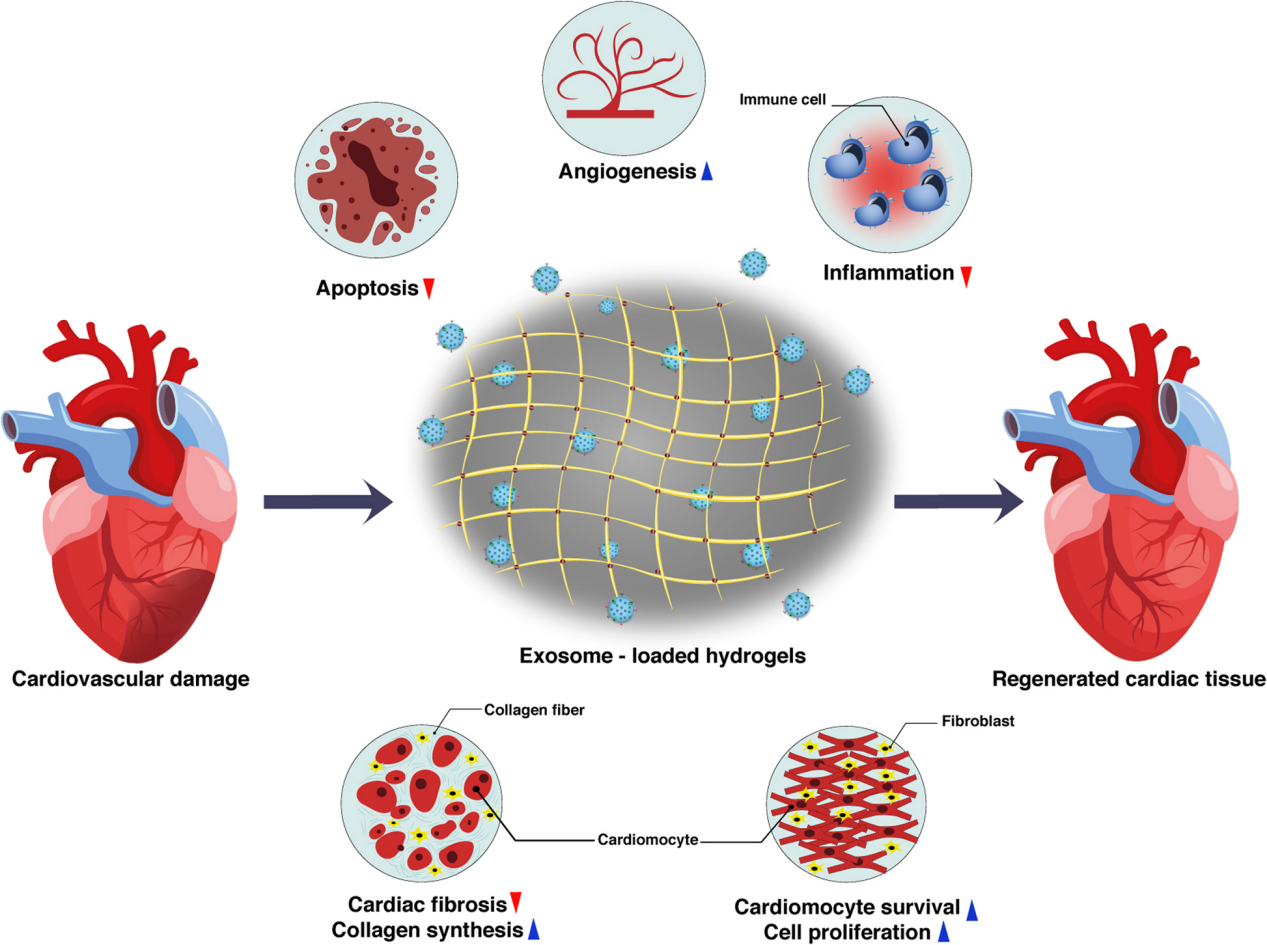
Exo-loaded hydrogels for cardiac tissue regeneration

Recently, Exos have gained the main focus in the diagnosis and treatment of cardiovascular diseases owing to their capability to reflect the physiological and pathological changes inside cardiac tissue [107]. Since Exos can circulate freely through the circulation system and are capable of transferring their bioactive cargo between cells, they play an important role in cell–to–cell communication, information transfer, and cell function and they act as a messenger between the heart and other organs, which improve tissue repair. The different miRNAs carried by Exos directly impact several aspects of cardiovascular diseases such as angiogenesis, hypertrophy, fibrosis, apoptosis, injury, and repair (Fig. 3) [108]. The use of biomaterials allows to modify their structure if needed so that they have properties suitable for the target tissue. Hydrogels with controllable degradation properties can play an important role in protecting Exos [101]. Therefore, not only the release profile of Exos loaded in hydrogel will be controllable but also this will create a suitable extracellular environment for the target cells to adhere, proliferate and migrate [33]. To this end, the selection of specific substrates and application of suitable processing modalities for the development of 3D hydrogels with adjustable mechanical values can preserve the function and morphology of encapsulated transplant cells after injection to the target sites [109]. Using surface modification strategies, it is possible to combine several natural and synthetic substrates to enhance the survival rate, proliferation, migration, and differentiation of cells [110]. According to previous experiments, natural substrates alone or in combination with synthetic materials with distinct cytocompatibility, degradation, and integration into cardiac tissue have been used for the regeneration of injured myocardium [111]. Hydrogels with suitable biocompatibility, hydrophilicity, compositional versatility, injectability, conductivity, and tunability are interesting platforms for mimicking cardiac ECM in in vitro and in vivo conditions [112]. Besides these features, hydrogels provide a favorable controllable delivery system depending on the type of substrates used in their structures. Hydrogels have been used for the regeneration of cardiac tissues by several therapeutic approaches [113]. They can be used as injectable substrates and administrated directly into the injured myocardium or by coronary artery perfusion [114]. Very recently, the application of hydrogels as cardiac muscle patches placed on the surface of injured areas with loaded cells, cytokines, drugs, therapeutics, and their combinations has been increased [115].

The advances in the technologies associated with the development of microgels, nanogels, and stimulus-responsive, adhesive, self-assembled injectable hydrogels have led to yielding better regenerative outcomes [116]. Along with these features, decellularized and electroconductive hydrogels have been at the center of attention for the healing of injured myocardium [117]. Along with the administration of cell-loaded hydrogels, Exo-loaded polymer-based hydrogels such as hyaluronic acid (HA), collagen, silk, gelatin, chitosan, fibrin, and alginate were also applied for the acceleration of cardiac tissue regeneration [118].

## Exo-loading strategies into hydrogels

Several studies have used naked EVs, especially Exos, for the restoration of injured cardiac tissue. Despite the acceleration of the healing process within the cardiac tissue after Exo injection, they are cleared from the target sites due to prolonged instability, heterogeneous delivery rates, and reactive phagocytic cells [119–122]. These data support the notion that a supporting niche is mandatory to increase the Exo therapeutic outcomes [123]. The stability and retention are important values for the application of Exos in in vivo conditions to yield better regenerative outcomes [1].

**Fig. 4:**
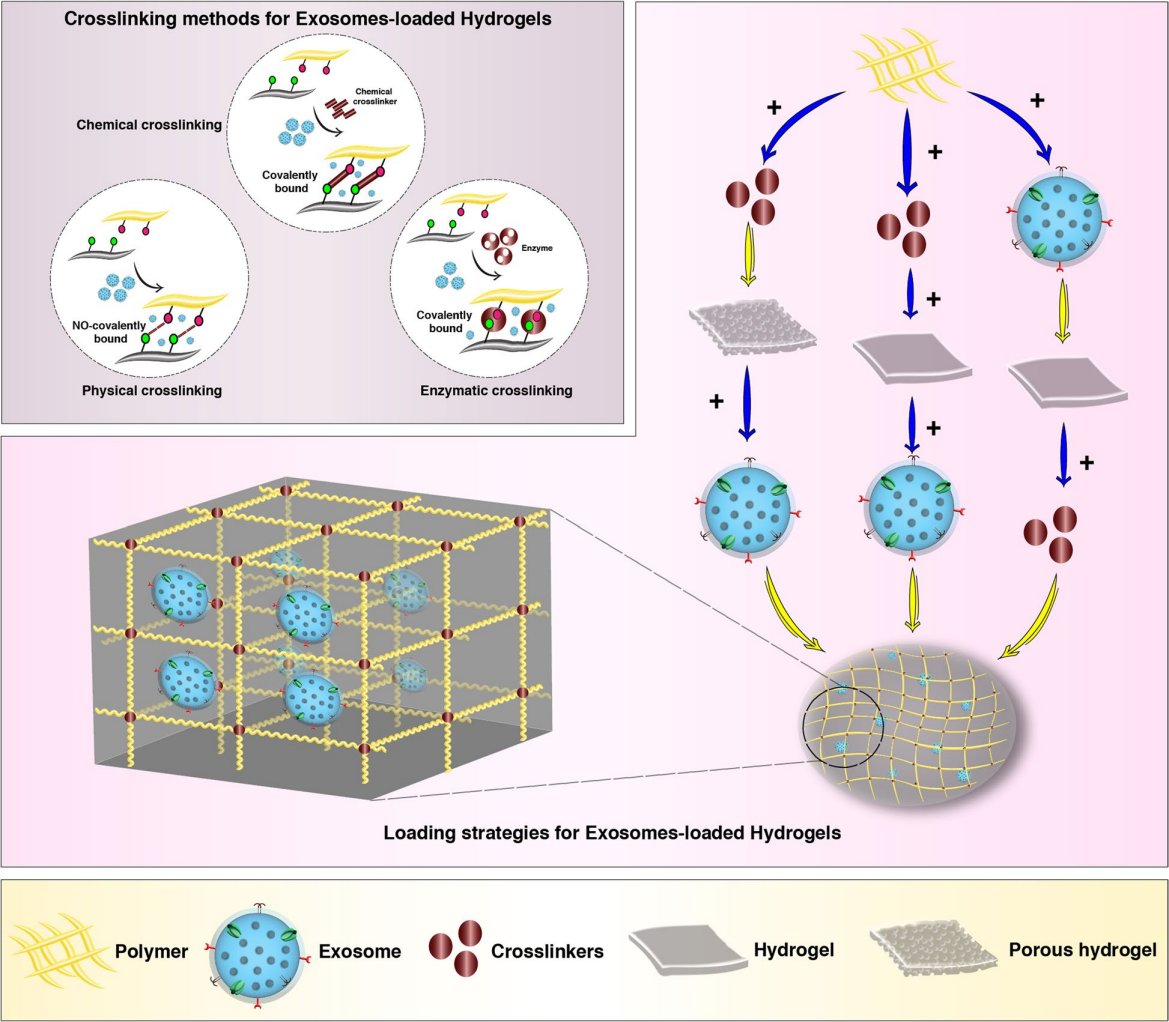
Common strategies used to encapsulate Exos inside the hydrogels. Hydrogel construction is done by the promotion of polymer-polymer interactions with chemical, physical, and enzymatic crosslinking approaches. Exos are integrated into the hydrogel before, during, or after the crosslinking process

Hydrogels are carrier platforms for controlled and sustained release of loaded Exos after introduction into the injured sites [29, 30]. Using an amalgamated system, hydrogels are the potential to support simultaneously the cell-to-cell interaction and release the loaded materials [111]. Hydrogels with passive hydrolytic degradation or cell-mediated enzymatic degradation, exhibit controllable degradation rate and can be used for optimized release of loaded Exos [32]. Currently, physical crosslinking and diffusion are the most commonly utilized strategy for Exo loading. In specific, the dispersion of Exos mainly is influenced by the pore size and crosslinking concentration of the hydrogel. Stronger interactions such as covalent connections and electrostatic interactions between the matrix and Exos can create more productive protection and immobilization of Exo [124]. According to the previous experiments, three main strategies are used to support Exo maintenance inside the hydrogels as follows [34];

- Exos and cross-linkers are mixed simultaneously with polymer-based hydrogels, and injected into the target sites using a dual-chamber syringe [33]. The promotion of gelation is promoted within the hydrogel using ion change, irradiation, and alteration of physicochemical values [35, 36]. It is thought that gelation can lead to suitable 3D conformation with irregular patterns while resulting in excellent integration and retention rates in the target sites [37–39]. The entrapping of CO_2_ bubbles increases the formation of interconnected porous networks within the hydrogels. These features help cell adhesion, migration, proliferation, and even ECM synthesis [40, 41]. A variety of mechanisms such as ion exchange, ultraviolet radiation, temperature changes, and pH changes can be used for in-situ gelation [125]. Data have suggested that this strategy is very important in filling the critical size defects of complex 3D shapes, allowing the combined biomolecules to have good viability. This type of injectable scaffold has the required inherent tissue properties, so it can work alone without external induction [126].
- Exos are mixed with polymers before the addition of cross-linkers. The process is continued with the addition of cross-linkers to yield gelous hydrogels. For example, Qin and co-workers used thiolated HA, gelatin, and heparin for the encapsulation of bone marrow stem cell (BMSC) Exos with the addition of PEG-diacrylate (PEGDA) as a cross-linker [42]. It should not be forgotten that strategies based on covalent crosslinking can delay the retention and release rates of encapsulated Exos within the hydrogels. Despite these advantages, the remnants of unreacted residual cross-linkers may be toxic. Under such circumstances, finding appropriate gelling temperatures and selecting non-toxic cross-linkers such as genipin are helpful [43–45].
- In the last strategy, the gelation of polymer is initiated with the addition of a cross-linker and before the Exo combination. In this method, swollen hydrogels are dehydrated followed by soaking in a solution containing Exos. It is postulated that super-water-absorbent and swelling capabilities potentiate the hydrogel to adsorb Exos and trap it in porous structures [46]. To this end, one could hypothesize that pore size is a fundamental factor in the fabrication of Exo-loaded hydrogels. Inappropriate porosity can lead to weak physical interpolation and excessive and earlier release of Exos (Fig. 4).

## Hydrogels as valid vehicles for Exo delivery to cardiac tissue

To date, various cell types have been used as a cell source for harvesting EVs however cargo type and quantity of isolated EVs can differ based on various factors such as tissue origin, sex, age, and environmental alternations [127]. Compared to somatic cells, stem cells exhibit higher capacities to release higher contents of EVs [128]. Therefore, it is logical to postulate that using tissue-derived stem cells such as cardiac stem cells can circumvent the factors affecting the quality of EVs [129]. Due to the ethical issues associated with the isolation, expansion, and cardiac stem cells, researchers tend to apply alternative cell populations for regenerative purposes. In an experiment conducted by Chen and co-workers, the rat endothelial progenitor cell (EPC) EVs were loaded onto injectable shear-thinning HA [9.33 × 10^9^/100 µl hydrogel] and injected into the border zone of ischemic cardiac tissue in a rat model of MI [130]. Based on the data, the infarct area was reduced in groups that received EV-loaded HA compared to the infarct group. Besides, EV-loaded HA induced angiogenesis by the increase of von Willebrand factor (vWF) and alpha-smooth muscle actin (α-SMA) cell density and CD11b cells in the peri-infarct [130]. They concluded that EV-loaded HA can improve the function of ischemic cardiac tissue by the promotion of angiogenesis and acceleration of ventricular geometry, leading to improved cardiac function.

Han et al. used self-assembled peptide amphiphile (PA) incorporated with cardiac protective peptides (GHRPS) and degradable amino acid sequence (GTAGLIGQ) for the delivery of encapsulated xenogeneic human umbilical cord MSC Exos in a rat model of MI [131]. To increase self-assembly properties, the progelator naphthalene acetyl tail was attached to the N terminus of the phenylalanine dipeptide. They found that the injection of 20 µl hydrogel containing 20 µg exosomal protein into the peri-infarct zone led to a reduction of fibrotic changes and down-regulation of TGF-β1 after 28 days [131]. Along with the reduction of apoptotic cardiomyocytes and increase of CD31 vascular cells, the number of CD68 inflammatory cells was reduced. Liu  and co-workers used encapsulated induced pluripotent-stem-cell-derived cardiomyocyte EVs (3 × 10^10^ particles) in collagen Gelfoam mesh (7 mm) for the alleviation of ischemic cardiac tissue injury in athymic nude rats [129]. Immunofluorescence data revealed an appropriate release of PKH-67 labeled EVs in the first 24 h after hydrogel patch transplantation. In infarct rats that received a hydrogel patch, the infarct size and pathological hypertrophy were diminished after 4 weeks, resulting in improved ejection fraction values.

**Fig. 5:**
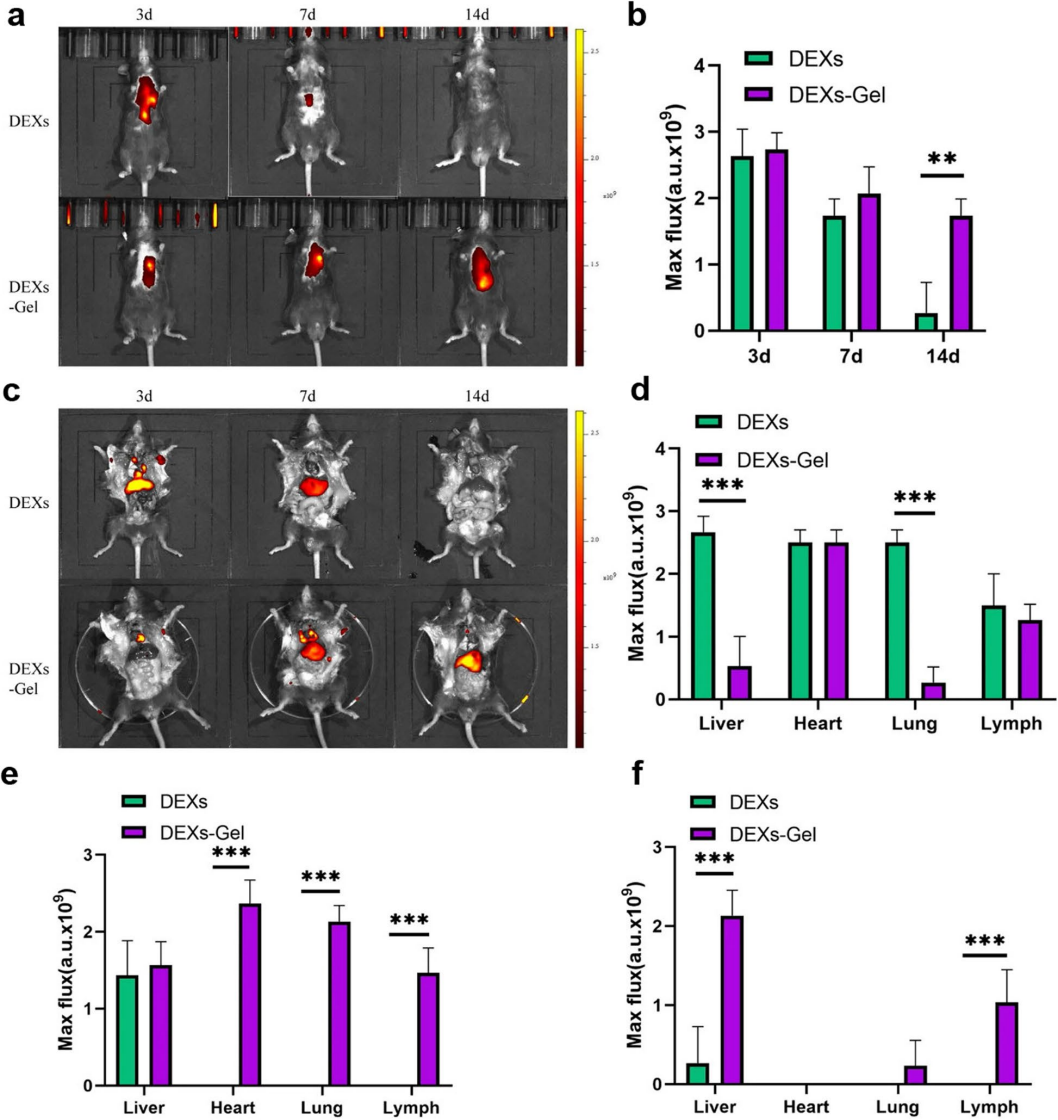
Alginate-based hydrogel is an appropriate platform for the sustained release of dendritic cell Exos (DEXs) into the targeted tissue over 14 days. Near-IR fluorescence indicated that the maximum flux values (a.u.) were high in mice that received DEX-loaded hydrogel compared to the DEX group after 14 days (**a**-**c**). Data indicated different a.u. values in several organs on days 3, 7, and 14 (**d**-**f**) (*n* = 3). Unpaired t-test. *p < 0.05, **p < 0.01, ***p < 0.001 [132]. Copyright 2021. Journal of Nanobiotechnology

It seems that the selection of EVs is an important factor in the control of inflammation and cell death rate in ischemic cardiac tissue along with the type and physicochemical properties of hydrogels [129]. It was also demonstrated that cardiomyocyte EV-loaded hydrogel patches can reduce the Caspase and TUNEL^+^ apoptotic cardiomyocytes in infarct myocardium compared to the groups with induced pluripotent stem cell EV-loaded hydrogel patches [129]. In a similar work, Zhang et al. used dendritic cell Exos loaded alginate hydrogel (30 µg exosomal protein/15 µl alginate hydrogel) for the regulation of immune cell response in an MI mouse model [132]. In an experiment, DEXs were labeled with DIR and incorporated inside the hydrogel composed of 2 and 1.5% (w/v) sodium and calcium alginate at a volume ratio of 1:1. DEXs alone and DEX-loaded hydrogel were injected into the ischemic region in mice model of MI. Based on the data, alginate hydrogel can preserve the loaded Exos for 14 days after injection into the infarct sites indicated by near-IR fluorescence images while in mice that received dendritic cell Exos, the injected particles were eliminated after 14 days post-infarct induction. The results indicated that incorporation within the hydrogels can last the stability and durability and presence of DEXs in in vivo conditions (Fig. 5) [132]. On days 5–7, Exo-loaded alginate hydrogel caused the reduced infiltration of iNOS^+^ M1 type macrophages into the injured sites. Along with these changes, the density of recruited CD206^+^ macrophages and Foxp3^+^ Treg cells was increased toward the ischemic region. Besides, the reduction of apoptotic cardiomyocytes and fibrotic changes were concurrent with enhanced regional vascular density (CD31^+^ cells).

### Advantages of hydrogels as factor delivery

**Fig. 6:**
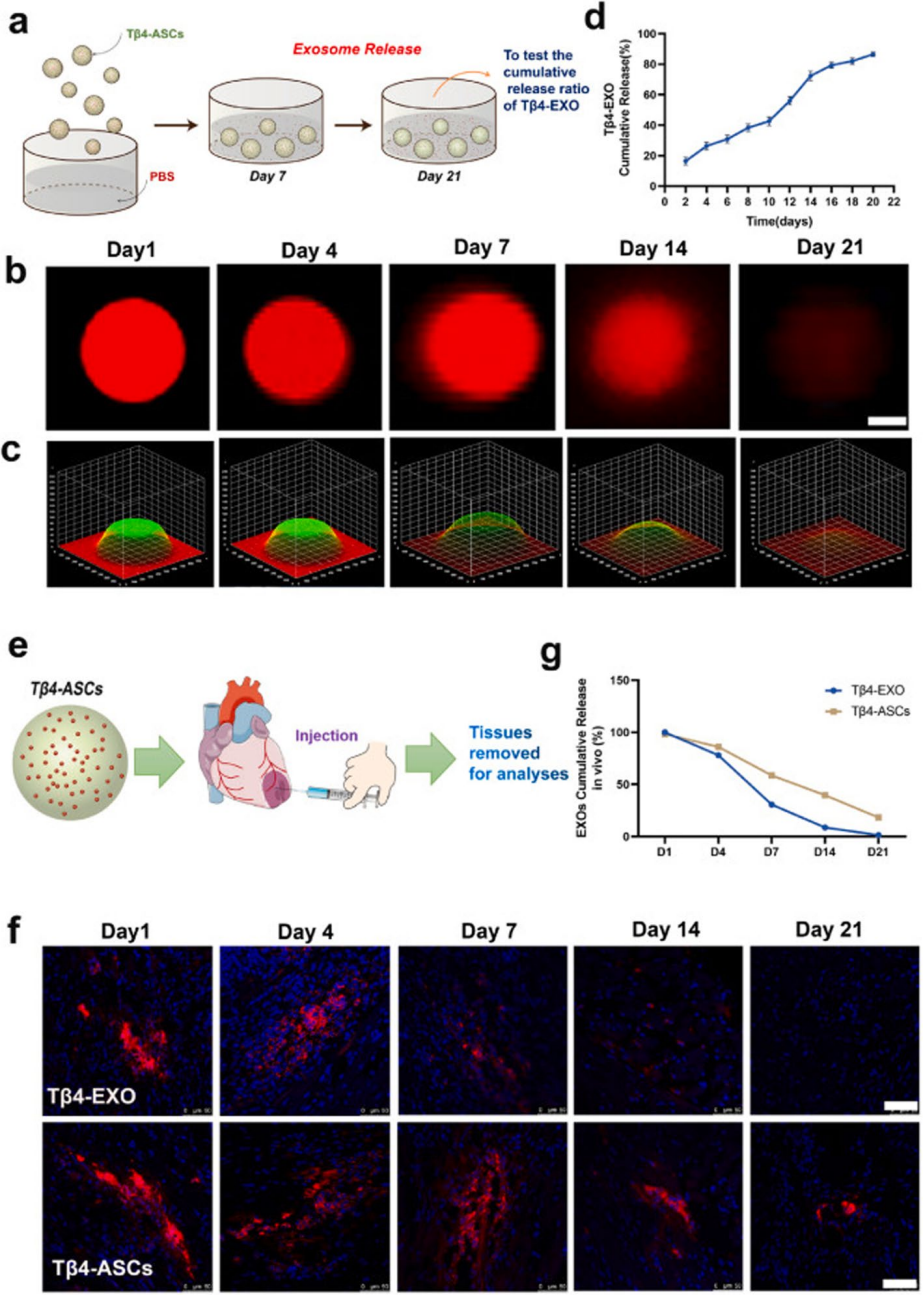
Hydrogels can release functional Exos for therapeutic purposes for long periods. The cumulative release of PKH-26-labeled Thymosin β4 (Tβ4)-Exos bearing GelMA/PEGDA microspheres has been documented for 21 days in in vitro conditions (**a**). Using confocal microscopy, fluorescence images at a 3D surface plot were prepared (**b** *-* **c**). Continuous delivery led to the reduction of fluorescence intensity inside the microspheres by increasing the release time. A fluorescence spectrophotometer indicates the cumulative release of PKH26-labeled Tβ4-Exos over 20 days after being injected into infarcted myocardium (**e**). Immunofluorescence images revealed an appropriate release of loaded Tβ4-Exos in in vivo conditions (**f**-**g**). Scale bar: 15 μm [135]. Copyright 2022. Bioactive Materials

**Fig. 7:**
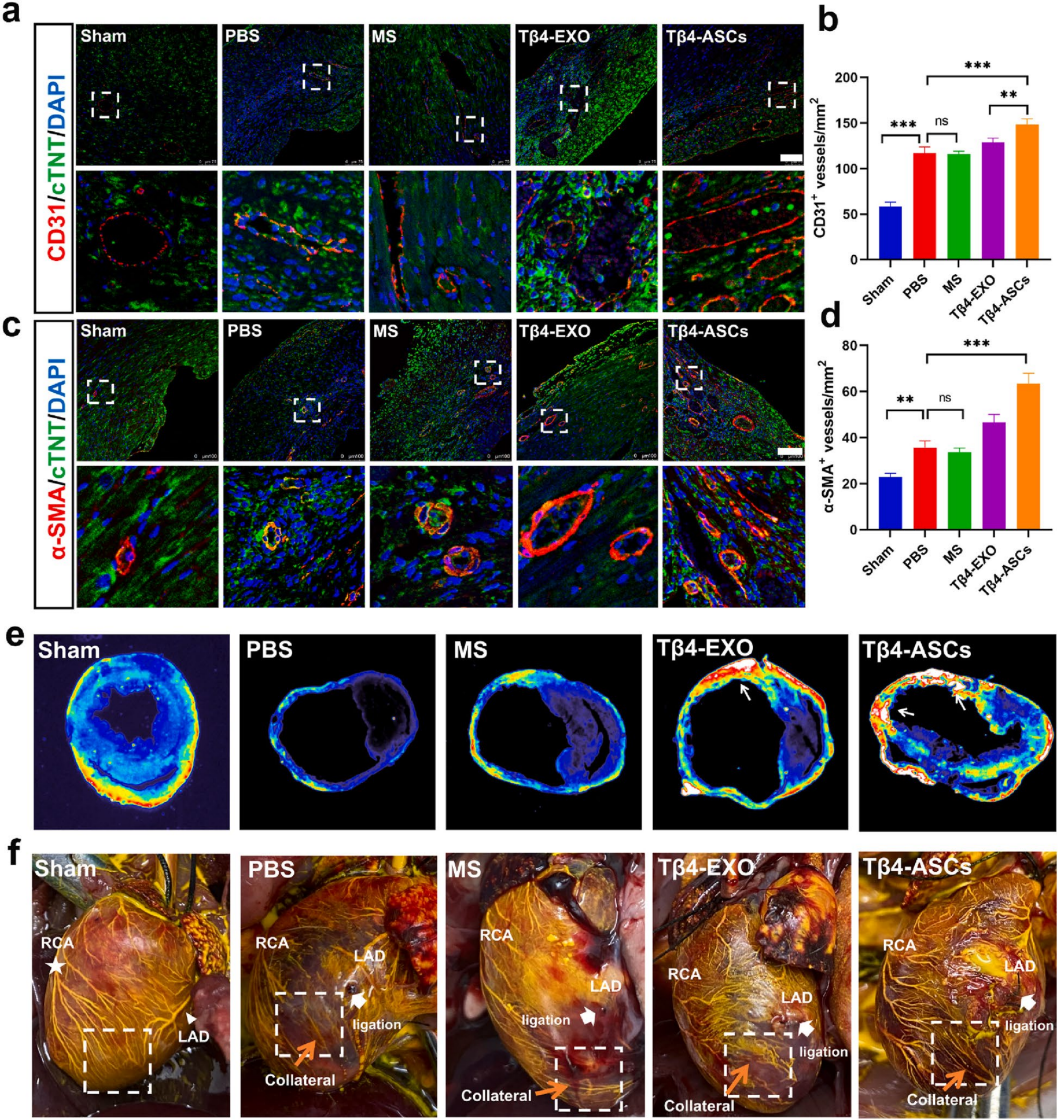
Exo-bearing hydrogels can be used for the induction of vascularization in ischemic changes. The angiogenic potential of Tβ4-Exos bearing GelMA/PEGDA microspheres was examined in a mouse model of MI (**a**-**f**). Using immunofluorescence staining, the capillary intensity (CD31^+^ vessels) was calculated in different groups including sham, infarct + PBS (PBS), GelMA/PEGDA microsphere alone (MS), Tβ4-Exos + GelMA/PEGDA microsphere (Tβ4-Exos), and Tβ4-Exo producing stem cells + GelMA/PEGDA microsphere (Tβ4-Exos + ASC) after 28 days (Scale bar: 75 μm) (**a** and **b**).The number of α-SMA^+^ arterioles (**c** and **d**). Cross-section of cardiac tissue and monitoring entire CD31 expression (**e**). The gross appurtenance of hearts after microfilling (**f**). White star: RCA (right coronary artery); White triangle: LCA (left anterior descending); White arrow: (the ligation of LAD). Microspheres (MS); Phosphate-buffered saline (PBS). One-way analysis of variance (ANOVA). **p < 0.01; ***p < 0.001 [135]. Copyright 2022. Bioactive Materials

In recent decades, the application of photopolymerizable 3D-printed gelatin methacrylate hydrogel has been increased in the tissue engineering era for the induction of angiogenesis in in vitro and in vivo conditions [133]. The placement of adipose MSC conditioned media-loaded gelatin methacrylate (GelMA) hydrogel increased the angiogenesis rate in aged cutaneous tissue via the modulation of the VEGF/Akt/mTOR and MAPK signaling pathway. It seems that Exo-loaded GelMA hydrogel can be also applied to the promotion of angiogenesis in infarct areas while physicochemical properties should be improved and adapted to the cardiac tissue microenvironment [134]. In this regard, Chen and co-workers fabricated GelMA/PEGDA [polyethylene (glycol) diacrylate] microsphere containing Thymosin β4 enriched Exos for induction of coronary collateralization in the MI mouse model [135]. The designed system was the potential to release the loaded Exos for 21 days in in vitro conditions. It was suggested that incubation of coronary artery endothelial cells (ECs) with Thymosin β4-Exo loaded GelMA/PEGDA microspheres increased the migration and tubulogenesis activity. Likewise, PKH26 labeled Thymosin β4-Exos were detected within the cardiac tissue about 21 days post-administration into the peri-infarct zone while direct injection of Thymosin β4-Exo into the injured myocardium without microspheres led to relatively rapid clearance rate (about 14 days) (Fig. 6) [135]. As expected, Thymosin β4-Exo loaded GelMA/PEGDA microspheres promoted the healing of injured myocardium via the induction of vascularization and reduction of apoptotic changes. In mice that received Exo-loaded microspheres, higher ejection fraction, and fraction shortening values were achieved compared to the other groups (Fig. 7) [135].

**Fig. 8:**
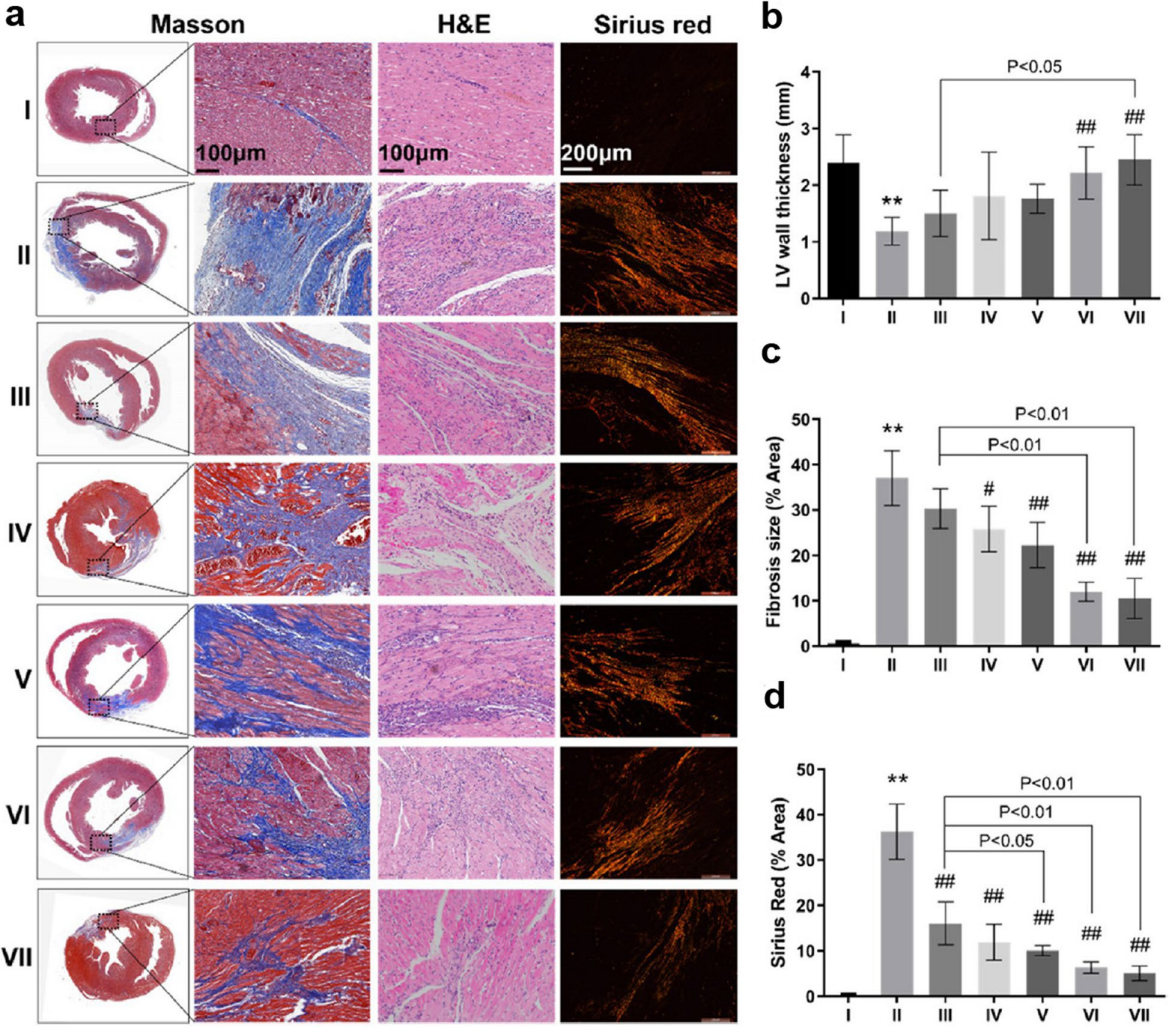
Measuring the cardiogenic potential of Exo-bearing conductive thiolated hyaluronic acid hydrogel in an ischemic/reperfusion model of rat after 28 days. Masson’s-trichrome, Hematoxylin-Eosin, and Sirius red staining (**a**). Left ventricle thickness (**b**), Fibrosis size (**c**), and Sirius Red positive area (**d**). (*n* = 3; **p < 0.01 versus Sham group; #*p* < 0.05 and ^##^ *p* < 0.01 vs. ischemic reperfusion model). (I: Sham; II: ischemic/reperfusion model; III: Free-Exo group; IV: EHBPE/HA-SH; V: AT-EHBPE/HA-SH; VI: AT-EHBPE/HA-SH@Exo; and VII: AT-EHBPE/HA-SH/CP05@. [136]. Copyright 2021. ACS Applied Materials & Interfaces

In an interesting study, HA was cross-linked with thiolated Exos anchoring a CP05 peptide using an epoxy/thiol click reaction. For this purpose, hyperbranched epoxy macromere and aniline tetramer were to entrap the human umbilical cord MSC Exos [136]. In vitro analyses indicated that the fabricated hydrogel possesses suitable electroconductive properties and appropriate shear-thinning injectability. It confirmed that the culture of HUVECs and HASMCs in the presence of HA-Exo-CP05 hydrogel enhances migration capacity. In line with these data, the viability of rat H9C2 cardiomyoblasts and mouse L929 fibroblasts was also induced. Injection of HA-Exo-CP05 hydrogel accelerates the healing of cardiac tissue after ischemia/reperfusion injury in a rat model by the reduction of fibrosis and induction of vascularization (CD31↑, α-SMA↑, VEGFA↑, VEGF-B↑, vWF↑) and cardiogenesis (Connexin-43↑, Ki67↑, SERCA2a↑) (Fig. 8).

To potentiate the angiogenic properties of encapsulated Exos inside the hydrogels, genetic manipulation of parent stem cells is a strategic approach to expedite vascularization and healing efficiency [137]. In support of this notion, Wang and colleagues claimed that encapsulated HIF-1α expressing MSC Exos inside the arginine-glycine-aspartate (RGD)-biotin hydrogel were appropriately uptaken by human ECs and rat cardiomyocytes. The exposure of rat cardiomyocytes to RGD-biotin hydrogel bearing HIF-1α-Exos prohibited the apoptotic changes (Caspases-3 and 7↓) in cardiomyocytes under normal and hypoxic conditions [137]. Under such conditions, the in vitro lumen formation properties of human ECs increased, indicating the functionality of encapsulated Exos. It was suggested that the existence of specific miRNAs especially miR-221 led to angiogenesis potential and survival activity in cardiomyocytes following the exposure to the insulting conditions. In a similar work, two angiogenic miRNAs (microRNA-126 and microRNA-146a) were loaded in Exos and encapsulated inside the alginate hydrogel. The injection of miRNA-bearing Exo with alginate promoted the angiogenesis and reduced fibrosis in a rat model of cardiac infarction [138]. Based on previous findings, it was found that the administration of RGD-biotin hydrogel bearing HIF-1α-Exos improved the heart function (ejection fraction↑ and fractional shortening↑) and increased the thickness of left ventricle by the reduction of type collagen I deposition [137].

It should not be forgotten that applied hydrogels should possess distinct physicochemical properties to provide timely the almost constant release of encapsulated Exos after introduction into the hypoxic cardiac niche. In this regard, Gil-Cabrerizo et al. encapsulated adipose MSC Exos inside the injectable hydrogel composed of 1% alginate, 0.5 mg/ml collagen, and 0.25% calcium gluconate [139]. Based on their findings, long-term retention with relative homogenous Exo release was proved after injection of alginate/collagen/gluconate hydrogel into the infarct myocardium in female rats. The highest retention was achieved in the group that received Exo-bearing hydrogel while the direct injection of naked Exos led to accumulation in non-specific organs such as the spleen and liver [139]. These data demonstrate that in situ administration of Exos is at least an effective approach to inhibit rapid exosomal clearance and trap in the reticuloendothelial system. The application of specific lineage Exos can also help us to dictate cardiomyocyte-like phenotype in transplanted stem cells and enhance cardiogenic potential [140]. In an experiment, cardiomyocyte Exos were encapsulated inside 3D HA hydrogel functionalized with tyramine and horse radish peroxidase (HRP) [140]. The culture of human MSCs with cardiomyocyte Exo (50 µl exosomal protein)-loaded HA (6%) hydrogel promoted cell differentiation into cardiomyocyte-like cells indicated with the up-regulation of Tbx5, GATA4, and Nkx2.5 [140].

## Injectable hydrogels loaded with Exo-like nanovectors

Like Exos, nanoparticles (NPs) are at the center of attention in terms of cardiovascular disease due to specific features such as surface energy, inherent surface topographies, and biological activities [141]. It has been indicated that effective delivery and long-term maintenance of Exo-like nanovectors can result in improving the regenerative capacity of cardiomyocytes [142]. Despite the existence of numerous therapeutic activities of Exos, it was suggested that heterogeneity, metabolic status of parent cells, and complexity of exosomal cargo can contribute to almost unpredictable reparative outcomes [143]. Besides, administrated Exos via systemic routes exhibit off-target delivery and are trapped in the liver, lungs, and spleen. Of note, repeated administration of Exos can also increase the risk of infection transmission, thrombosis, and asphyxiation [144]. The fluctuation of zeta potential is an important factor that affects the stability of Exos while in synthetic NPs zeta potential values vary making them more stable [145].

In an experiment, Yang and co-workers synthesized polymeric NPs composed of a fluorescent PFBT (polymer based on fluorene and benzothiadiazole) core, 1,2-Distearoyl-sn-glycero-3-phosphoethanolamine-Poly (ethylene glycol (DSPE-PEG) shell with attached miR-199a-3p and penetrating amino acid TAT for induction of angiogenesis in cardiac tissue [146]. The NP structure was incorporated inside the cross-linked elastin-like protein (1%)-HA (1%) and injected into the ischemic site in the rat MI model [146]. It confirmed that about 90% of loaded miRNA was released into the ischemic site in the presence of MMP-9 after 5 days post-injection into the cardiac tissue, increasing angiogenesis, cardiac output, and reduction of scar tissue. In an experiment, mesoporous silica NPs (MSNs) are loaded with miR-21-5p and combined with a pH-responsive hydrogel, and injected into Yucatan mini pigs MI model [147]. This strategy accelerated the regeneration of ischemic cardiac tissue by simultaneous inhibition of inflammation and promotion of angiogenesis.

More recently, self-healing and shear-thinning hydrogels have been developed as a promising platform for therapeutic cargo delivery. These hydrogels can be injected into the target sites via minimally invasive surgical approaches [148]. In this regard, a versatile and unique nanotechnology-based drug delivery platform with self-healing and shear-thinning properties containing PEG and silicate nanodisks, as an Exo-like nanovector, was fabricated for in situ delivery of dexamethasone into the epicardial surface [149]. Data indicated the suppression of inflammatory response in a rabbit model of epicardial injury with the reduction of CD3^+^ and CD68^+^ and down-regulation of NF-κB [149]. Likewise, the treatment of ECs with designed silicate nanodisks led to the inhibition of inflammatory surface molecules such as ICAM-1 and VCAM-1, and TNF-α in in vitro settings [149]. Chen and co-workers fabricated an injectable and self-healing elastin-mimic hydrogel carrying salvianolic acid B-loaded polydopamine NPs for the regeneration of cardiac tissue in a rat MI model [150]. Micro-CT scanning revealed the stability of injected hydrogel at the site of ischemia even after 20 days. Histological data confirmed the reduction of TUNEL^+^ cardiomyocytes and the increase of vWF^+^ and α-SMA^+^ vessels in the peri-infarct zone [150].

**Fig. 9:**
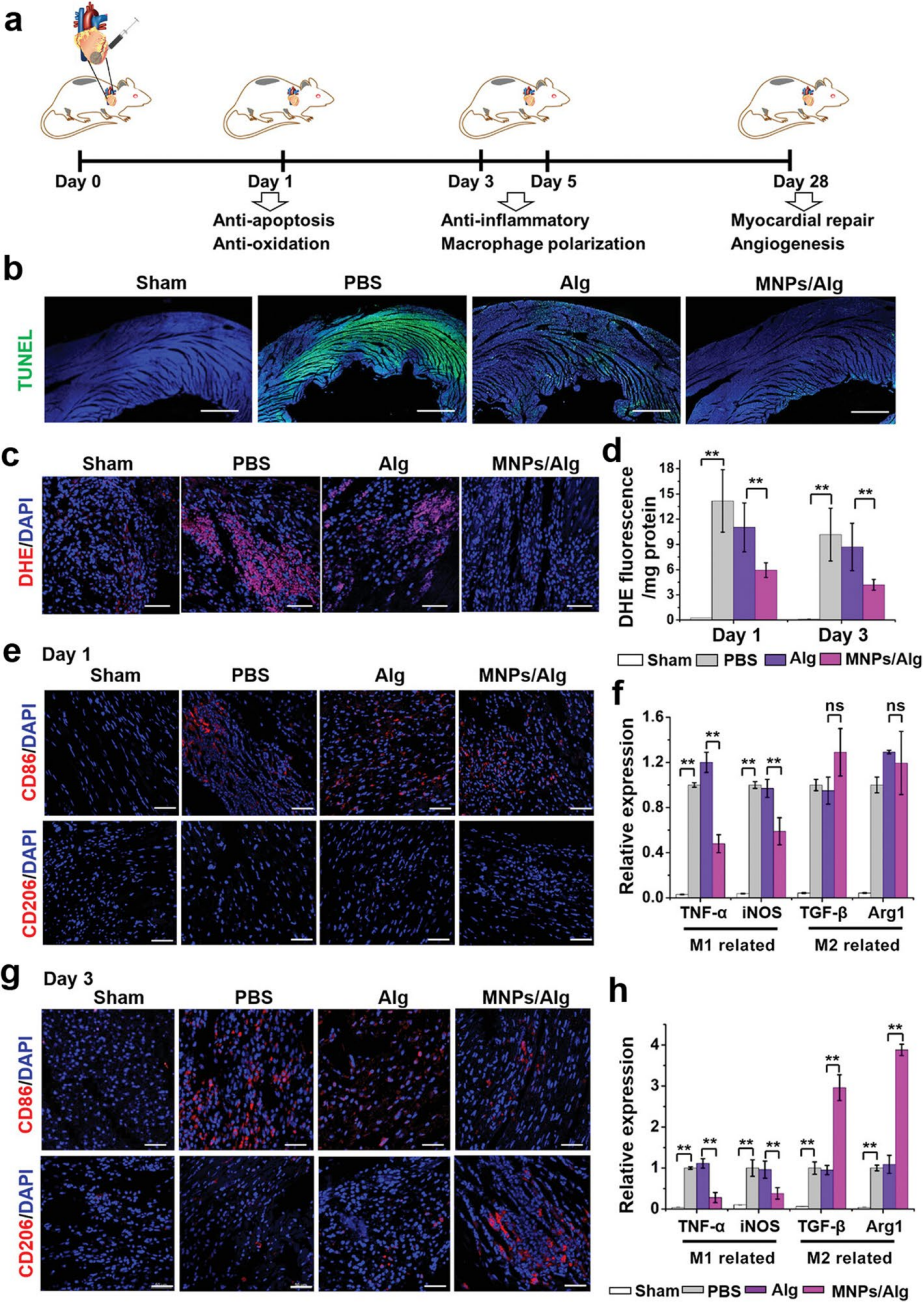
Monitoring regenerative potential of melanin nanoparticle-loaded alginate hydrogel (MNPs/Alg hydrogel) on reactive oxygen species (ROS) and immune response in a rat model of MI. A time-dependent healing process occurred within the ischemic area after MNPs/Alg hydrogel transplantation (**a**). Detection of apoptotic cells in the infarcted zone using TUNEL assay 1-day post-hydrogel administration (**b**). Immunofluorescence images related to O2•– levels using DHE staining 1 day after hydrogel injection (**c**). Detection of O2•– levels using ROS detection kit (DHE) on days 1 and 3 (**d**). The levels of CD86 and CD206 positive macrophages within the infarct zone were visualized using immunofluorescence images (**e**). Expression of pro-inflammatory (TNF-α and iNOS), and anti-inflammatory (TGF-β and Arg1) cytokines 1 day after injection (**f**). CD86 and CD206 positive macrophages after 3 days (**g**). Expression of pro-inflammatory, and anti-inflammatory cytokines on day 3 (*n* = 3). Scale bar: 50 μm; Student’s t-test. *p < 0.05, **p < 0.01; and nsp > 0.05 [153]. Copyright 2021. Advanced Science. 2021

The acceleration of tissue modeling is a strategic approach to expedite cardiac tissue regeneration after injuries. In this regard, Wei and colleagues fabricated MMP-sensitive and conductive hydrogel using oxidized alginate incorporated with tetraaniline. The hydrogel was loaded with nano-drug 1, 4-dihydrophenonthrolin-4-one-3-carboxylic acid (DPCA) synthesized using polymerized dopamine. The oxidized alginate was cross-linked with thiolated HA and MMP-sensitive peptide [151]. The injection of prepared hydrogel enhances regulation in rat ischemic heart by the suppression of inflammation (Caspase 3↓, and TNF-α↓) and reduction of fibrosis. Along with these changes, the protein levels of troponin T, and connexin-43 were induced, indicating the cardiogenic potential of developed hydrogel [151]. The scavenging of accumulated reactive oxygen species at the site of ischemia can help the injured cardiomyocytes to restore their function [152]. Using melanin NPs inside the alginate hydrogel, Zhou and co-workers investigate the cardiogenic potential of the designed hydrogel in a rat MI model [153]. The injection of melanin NP-loaded alginate hydrogel promoted the polarization of macrophages toward the M2 phenotype (CD206↑, CD68↓) and increase cardiomyocyte resistance against oxidative stress induced by oxygen peroxide (Fig. 9) [153].

Stromal cell-derived factor 1 (SDF-1α) is known to play a central role in stem cell homing, retention, survival, proliferation, cardiomyocyte repair, angiogenesis, and ventricular remodeling following MI. It acts as the unique ligand for its receptor CXCR4 and the SDF-1–CXCR4 axis is up-regulated in both experimental and clinical studies of MI [154]. Transient delivery of SDF-1α either through the delivery of protein-encoding genes or the protein itself has demonstrated improved cardiac function in preclinical models [154]. However, poor retention when injected into the contracting myocardium and fast protein degradation, which needs prolonged activity to be therapeutically effective are restrictions of myocardial regeneration therapy with this method. To address these limitations, catheter-based injectable gels have been designed to deliver SDF-1α encoding minicircles (MC). HA, and recombinant elastin-like protein have been used for the synthesis of this biocompatible gel with shear-thinning and self-healing capacities for in situ delivery of SDF-1a-encoding MC in rat MI model [155].

## Advantages and limitations of Exos for cardiac tissue

In recent years, the development of hydrogel-bearing Exos as a *de novo* delivery system has been at the center of attention in restoring and improving the function of cardiac tissue with MI [156]. According to a plethora of scientific documents, direct injection of Exos directly without supporting hydrogel can lead to the rapid elimination of these nanoparticles before enhancing cardiomyocyte proliferation and regeneration [51]. Such limitations in the effective delivery of Exos to target sites have yet to be overcome [157]. Hydrogels can provide an ideal microenvironment to protect the integrity of Exos against harsh in vivo conditions with long-term release into tissue following injection [33]. Besides, the physicochemical properties of hydrogels can be modulated by varying their constituent components [158]. To prepare targeted Exo-loaded hydrogels, the influence of some factors such as the swelling rate, surface charge, porosity, and degradation rate of hydrogels on the loading and release of Exos should be carefully analyzed. The loose and porous network structure of hydrogel is one of the most significant requirements for achieving efficient loading and prolonged release of Exos [159]. The concentration of polymer, cross linker and external stimuli such as light, pH, and temperature should not be neglected [160]. The large pores permit Exos to be easily loaded into the hydrogel, but cause Exos to be released in a cascade way, which often does not efficiently delay Exo retention time. For hydrogels with very small pores, the release of Exo is dependent on the swelling rate and degradation capacity [161]. Using smart/stimuli-responsive hydrogels, the release of Exos can be regulated by altering the swelling rate in the presence of some environmental variables such as temperature, pH, and light [162].

**Fig. 10:**
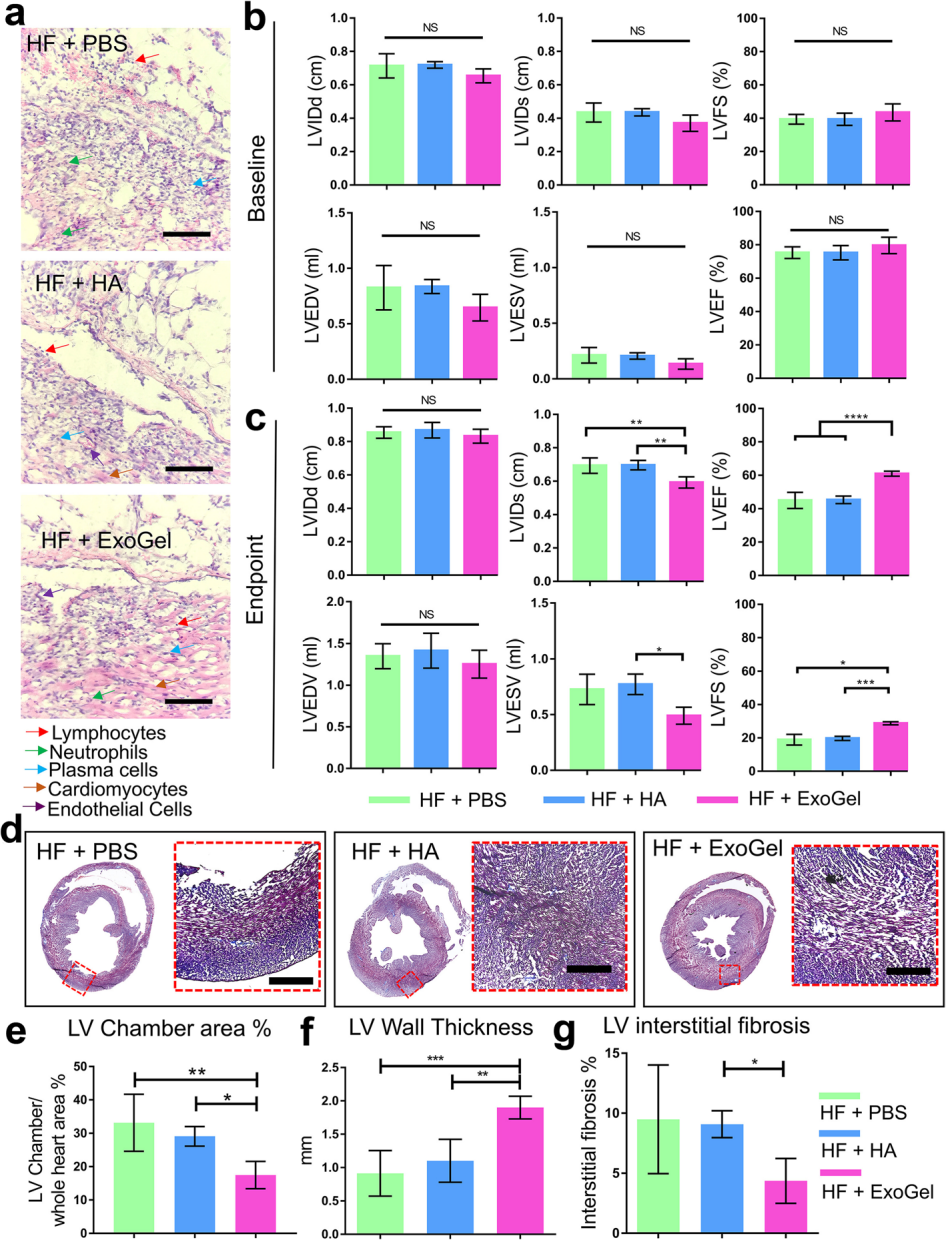
Regenerative potential of Exo-loaded hyaluronic acid hydrogel in a rat model of transverse aortic constriction (TAC). H & E staining was performed 28 days after hydrogel administration (**a**; Scale bar: 200 μm). Different functional parameters, including were LVIDd, LVIDs, LVFS, LVEDV, LVESV, and LVEF were measured using echocardiography before TAC (**b**) and 28 days after TAC induction (**c**). The levels of fibrotic changes were evaluated using Masson’s Trichrome staining in rats that received PBS, hydrogel alone (HA), and Exo-loaded hydrogel (ExoGel) 28 days after transplantation (**d**; Scale bar: 400 μm). LV chamber area (**e**; *n* = 5); LV wall thickness (**f**; *n* = 5); Interstitial fibrosis rate (**g**). One-Way ANOVA analysis with post hoc Bonferroni test. *p < 0.05; **p < 0.01; ****p < 0.0001. HA: heart failure; LV: left ventricle; LV internal diameter end diastole: LVIDd; End-systole: LVIDs; LV fractional shortening: LVFS; LV end-diastolic volume: LVEDV; LV end-systolic volume: LVESV; and LV ejection fraction: LVEF. Copyright 2022 [165]. Journal of Molecular and Cellular Cardiology

As previously mentioned, several forms of hydrogels and crosslinking agents with different mechanisms have been exploited to develop Exo-loaded substrates in the field of cardiovascular tissue engineering [34]. However, challenges associated with the potential toxicity of chemical cross-linkers and possible detrimental effects on Exo integrity remain to be answered [163]. Besides, in situ application of injectable hydrogels fabricated using temperature- or pH-, ionic, and photo-based cross-linking methods should be carefully examined. Values such as polymer concentration, gelation temperature, and applicator system should be considered before the injection of Exo-bearing hydrogels [164]. Despite the existence of regenerative properties, the administration of Exo-bearing hydrogels faces some limitations that restrict their application in ischemic cardiac tissue [165]. Exo-bearing hydrogel delivery is done via invasive approaches such as intricate surgical processes and heart manipulation [165]. Therefore, the advent and development of less invasive approaches seem critical. Cardiac patches composed of natural and/or synthetic substrates can provide a reliable delivery platform for highly efficient Exo delivery into the infarct myocardium [165]. In this scenario, Chen and co-workers loaded MSC Exos onto the HA substrate (1 × 10^9^ particles per ml of hydrogel) and injected via the epicardial side of rat hearts with aortic constriction injury. Data indicated appropriate retention and biodistribution of loaded Exos toward the injured myocardium led to a reduction of fibrotic changes (Fig. 10) [165].

## Conclusion

Since Exos exhibit various regenerative properties in terms of cardiovascular diseases, especially in ischemic myocardial disease, attempts should be directed toward the advent and development of *de novo* modalities for improving therapeutic outcomes. It is thought that Exos employed in cell-free therapies are compromised by off-target effects and rapid elimination action. Thus, the application of hydrogels and 3D-engineered tissue matrices with Exos provides a suitable microenvironment to postpone Exo retention time in the damaged cardiac. However, there is still an avenue for researchers to optimize future approaches and expand studies to confirm the efficacy and safety of these promising approaches and ensure scientific and technology transfer to the appropriate clinical setting.

## Data Availability

Not applicable.

## Abbreviations

DSPE-PEG: 1, 2-Distearoyl-sn-glycero-3-phosphoethanolamine-polyethylene glycol
DPCA: 1, 4-dihydrophenonthrolin-4-one-3-carboxylic acid
α-SMA: Alpha-smooth muscle actin
RGD: Arginine-glycine-aspartate
BMSCs: Bone marrow stem cells
ESCRT: Endosomal sorting complexes required for transport
ECs: Endothelial cells
EPCs: Endothelial progenitor cells
Exos: Exosomes
ECM: Extracellular matrix
EVs: Extracellular vesicles
GelMA: Gelatin methacrylate
HRP: Horse radish peroxidase
HA: Hyaluronic acid
ILVs: Intraluminal vesicles
MSCs: Mesenchymal stem cells
MSNs: Mesoporous silica NPs
MC: Minicircles
MVBs: Multivesicular bodies
MI: Myocardial infarction
NPs: Nanoparticles
PEGDA: PEG-diacrylate
PA: Peptide amphiphile
PFBT: Polymer based on fluorene and benzothiadiazole
SNARE: Soluble NSF Attachment Protein Receptor
SDF-1α: Stromal cell-derived factor 1
3D: Three dimensional
vWF: von Willebrand factor
